# Serum levels of tumor necrosis factor alpha in patients with IgA nephropathy are closely associated with disease severity

**DOI:** 10.1186/s12882-018-1069-0

**Published:** 2018-11-14

**Authors:** Guanhong Li, Wei Wu, Xinyao Zhang, Yuan Huang, Yubing Wen, Xuemei Li, Ruitong Gao

**Affiliations:** 1 Division of Nephrology, Department of Internal Medicine, Peking Union Medical College Hospital, Chinese Academy of Medical Sciences & Peking Union Medical College, NO.1, Shuaifuyuan, Dongcheng District, Beijing, 100730 China; 2 Department of Clinical Laboratory, Peking Union Medical College Hospital, Chinese Academy of Medical Sciences & Peking Union Medical College, Beijing, China

**Keywords:** Biomarkers, Cytokines, Tumor necrosis factor alpha, IgA nephropathy

## Abstract

**Background:**

Tumor necrosis factor alpha (TNF-α) is considered to play an important role in the pathogenesis in IgA nephropathy (IgAN). The correlations between serum TNF-α and disease severity in patients with IgAN remain controversial.

**Methods:**

Concentrations of serum TNF-α of 147 patients with IgAN and 126 healthy subjects were measured by chemiluminescence immunoassay. Correlations with clinicopathological features of patients with IgAN were evaluated.

**Results:**

Serum levels of TNF-α [9.20 (7.70–10.60) pg/mL vs. 6.04 (5.11–7.23) pg/mL, *P* < 0.0001] were higher in patients with IgAN than that in healthy subjects. Receiver operating characteristic curve analysis revealed that TNF-α had better discrimination between patients with IgAN and healthy controls than estimated glomerular filtration rate [TNF-α: (AUC, 0.87; 95% CI, 0.83–0.91; *P* < 0.0001) vs. estimated glomerular filtration rate: (AUC, 0.76; 95% CI, 0.71–0.82; *P* < 0.0001), *P* = 0.007]. Multivariate linear regression analyses showed that serum levels of TNF-α were positively correlated with 24-h urine protein excretion (*r* = 0.33, *P* = 0.04), urinary protein to serum creatinine ratio (*r* = 0.33, *P* = 0.03), serum creatinine (*r* = 0.46, *P* < 0.0001) and Cystatin C (*r* = 0.59, *P* < 0.0001) in IgAN and negatively correlated with estimated glomerular filtration rate (*r* = − 0.49, *P* < 0.0001) after adjustment for sex, systolic blood pressure and diastolic blood pressure. Patients with higher mesangial hypercellularity or tubular atrophy/interstitial fibrosis score according to Oxford classification showed higher serum levels of TNF-α.

**Conclusions:**

Our data showed that serum levels of TNF-α detected by chemiluminescence immunoassay was a potential biomarker for evaluating the disease severity in IgAN.

**Electronic supplementary material:**

The online version of this article (10.1186/s12882-018-1069-0) contains supplementary material, which is available to authorized users.

## Background

IgA nephropathy (IgAN) is the most common cause of primary glomerulonephritis [1]. IgAN is associated with a poor prognosis, about 20–40% of patients with IgAN progress slowly to end-stage renal failure 20–25 years after diagnosis [2, 3]. Heavy proteinuria, hypertension, renal dysfunction, and histological features recently updated Oxford classification have been identified as clinicopathological markers for disease severity and poor prognosis of IgAN [4, 5]. However, these factors are numerous and controversial, and do not always have the specificity to identify an individual prognosis [2, 4]. Recently, several biomarkers such as serum galactose-deficient IgA1 and the corresponding autoantibodies [6, 7], soluble CD89 levels [8], urinary soluble transferrin receptor [9], urinary interleukin-6/epidermal growth factor (IL-6/EGF) [10] and other serum and urine biomarkers are associated with histological findings of severity and poor outcomes in IgAN. However, the utility of these biomarkers is not yet well defined.

It is considered that cytokines such as tumor necrosis factor-α (TNF-α) play an important role in aberrant mucosal immune response in the early phase of the pathogenesis in IgAN [11, 12], and mesangial cell proliferation, hyperproduction of extracellular matrices, podocyte injury and glomerulosclerosis in the second phase [13]. Furthermore, our recent study showed that hydroxychloroquine (HCQ) targeting cytokines including TNF-α was effective in ameliorating proteinuria [14]. Therefore, serum TNF-α may be a potential biomarker for severity in IgAN.

However, the studies concerning serum levels of TNF-α as a biomarker in IgAN were inadequate. The relationship between serum TNF-α levels and clinical parameters, pathological features had not been fully established in IgAN [15, 16].

Concerning the detection method of TNF-α, enzyme linked immunosorbent assay (ELISA) is still recommended. Recently, because of the high sensitivity, rapidity of reaction, simple instrumentation, and wide dynamic range, fully automated chemiluminescence immunoassay (CLIA) has been used as an attractive method in different fields, such as biotechnology, pharmacology, molecular biology [17, 18]. And CLIA has also been used to detect TNF-α in the clinical laboratory of our hospital.

Therefore, in this study, we investigated the value of serum levels of TNF-α in a large sample of patients, detected by CLIA with higher sensitivity and better stability, aiming to explore the role of TNF-α in evaluating disease severity in IgAN.

## Methods

### Patients

Serum samples were collected from 147 patients with biopsy-proven primary IgAN between July 2016 and April 2017. The patients accepted renal biopsy between November 1999 and December 2016. In addition, 126 serum samples were collected from healthy volunteers who had no known kidney diseases between August 2015 and February 2016. Clinical and laboratory data were collected at the time of cytokines measurement in the clinical laboratory of Peking Union Medical College Hospital. Patients with lupus nephritis, Henoch–Schönlein purpura, liver cirrhosis and other secondary causes of IgAN were excluded from the study. Patients with concomitant systemic disease, autoimmune diseases, neoplastic diseases and infection which might cause abnormal elevation of serum TNF-α or IL-6 were also excluded. All patients gave written informed consent to the study, which had received Local Ethical Committee approval.

### Clinical and pathological features

Full medical histories and physical findings were documented. The demographic and the clinical parameters of patients including age, gender, blood pressure (BP), urinary red blood cells count, urine protein/creatinine ratio, 24-h urine protein excretion were recorded. Blood chemistry tests included serum creatinine, Cystatin C and IgA. Estimated glomerular filtration rate (eGFR) was calculated using the Chronic Kidney Disease Epidemiology Collaboration (CKD-EPI) equation [19]. Chronic Kidney Disease (CKD) stages 1–5 were divided by eGFR≥90 (G1), 60–89 (G2), 45–59 (G3a), 30–44 (G3b), 15–29 (G4), and < 15 (G5) mL/min/1.73 m^2^, respectively, according to the new KDIGO (Kidney Disease: Improving Global Outcomes) classification [20]. Histologically, the updated Oxford classification was used for evaluating the pathological lesion [5].

Medication history, including the usage of cortisone, immunosuppressant and renin-angiotensin system blockers such as angiotensin-converting enzymes inhibitors (ACE-Is) and angiotensin II receptor blockers (ARBs) was also recorded.

### Measuring serum TNF-α

Blood samples were obtained from each patient after an overnight fast upon admission. The blood samples were allowed to clot at room temperature for 30 mins and centrifuged for 10 minutes at 3000 rpm. The serum samples were separated as soon as possible from the clot of red cells after centrifugation to avoid TNF-α production by blood cells that falsely could increase its values. Separated sera were measured immediately. Levels of serum TNF-α were measured in the clinical laboratory at Peking Union Medical College Hospital using Tumor necrosis factor-alpha Assay Kit (chemiluminescent assay) through IMMULITE® 1000 system (Siemens Healthcare Diagnostics Inc., United Kingdom) according to the manufacturers’ instructions. Detection limit of the kits was obtained from the manufacturers, as follows: TNF-α (detection limit 1.7–1000 pg/mL). The recommended reference range was as follows: TNF-α (≤ 8.1 pg/mL).

### Statistical analysis

Statistical calculations were performed using SPSS software for Windows, version 20.0 (SPSS Inc., Chicago, IL) and Graph Pad Prism 5.0 (Graph Pad Software Inc., San Diego, CA). Data are presented as medians (interquartile range) or frequency in percent according to the types of variables. The Mann–Whitney U-test was used for statistical comparisons between two groups, and the Kruskal-Wallis Test was used for statistical comparisons among more than two groups. Categorical variables were compared using chi-squared test. To set the cut-off values between IgAN patients and healthy controls (HC), we used the receiver operating characteristic (ROC) curve analyses to find the best compromise value between sensitivity and specificity; we also calculated area under the curve (AUC) with 95% confidence interval (CI) and *P* values. Serum levels of TNF-α were subjected to logarithmic transformation before correlation analyses. Bivariate correlation analyses were performed using Spearman’s correlation analysis. Multivariate linear regression analyses adjusted for sex, systolic BP and diastolic BP were performed to examine the correlations between serum levels of TNF-α and clinical parameters. All tests were two-sided and a *P*-value < 0.05 was considered statistically significant.

## Results

### Demographic and clinical features in the IgAN patients and healthy subjects

In our study, 147 patients with IgAN [mean age, 35 (29–45) years; male/female ratio, 71/76] and 126 healthy subjects [mean age, 40 (30–49) years; male/female ratio, 61/65] were involved. The main demographic, clinical and the pathological features of patients with IgAN were summarized in Table 1. The average levels of BP, 24-h urine protein excretion, and eGFR were 120 (110–130) / 75 (70–80) mmHg, 0.82 (0.38–1.55) g/24 h, 79.29 (60.00–104.29) mL/min•1.73m^2^, respectively. In this study, 35 (23.8%) IgAN patients were untreated, 99 (67.3%) were treated with ACE-Is/ARBs, 71 (48.3%) were treated with oral corticosteroids, and 76 (51.7%) were treated with other immunosuppressive agents at the time of sample collection. The detailed demographic and clinical data of healthy subjects were showed in Additional file 1.

**Table 1:** Demographic, clinical and pathological features of patients with IgA nephropathy

Characteristics	Values
Patients numbers	147
Cytokines–biopsy time interval^a^ (months)	27.93 (6.17–63.37)
Mean age (yr)	35 (29–45)
Male, n (%)	71 (48.3)
Systolic BP (mmHg)	120 (110–130)
Diastolic BP (mmHg)	75 (70–80)
Mean arterial pressure (mmHg)	90 (83–97)
Urinary red blood cell (/μL)	29 (7–79)
Urinary protein to serum creatinine ratio (mg/g Cr)	595 (244–1305)
24-h urine protein excretion (g/24 h)	0.82 (0.38–1.55)
< 0.3	27 (18.4%)
0.3–0.99	59 (40.1%)
1.0–2.99	46 (31.3%)
≥ 3	15 (10.2%)
Serum creatinine (mg/dL)	1.07 (0.84–1.39)
Serum IgA (g/L)	2.22 (1.82–3.22)
Cystatin C (mg/dL)	1.06 (0.93–1.57)
eGFR (mL/min•1.73m^2^)^b^	79.29 (60.00–104.29)
CKD Stages^c^, n (%)	
1	57 (38.8)
2	49 (33.3)
3a	22 (15.0)
3b	8 (5.4)
4	5 (3.4)
5	6 (4.1)
Oxford classification^d^, n (%)	
M0/ M1	16(10.9), 131(89.1)
E0/E1	113(76.9), 34(23.1)
S0/S1	24(16.3), 123(83.7)
T0/T1/T2	45(30.6), 65(44.2), 37(44.2)
C0/C1/C2	66(44.9), 66(44.9), 15(10.2)
Medical treatments, n (%)	
Untreated	35 (23.8)
Corticosteroids	71 (48.3)
Immunosuppressants	76 (51.7)
ACE-Is/ARBs	99 (67.3)

Data are presented as median (interquartile range) or frequency in percent

*BP*: blood pressure; 1 mmHg = 0.133Kpa; *eGFR*: estimated glomerular filtration rate; *ACE-Is*: angiotensin converting

enzyme inhibitors; *ARBs*: angiotensin II receptor blockers. *M*: mesangial hypercellularity score < 0.5 (M0) or > 0.5 (M1);

*E*: endocapillary hypercellularity absent (E0) or present (E1); *S*: segmental glomerulosclerosis absent (S0) or present (S1),

presence or absence of podocyte hypertrophy/tip lesions in biopsy specimens with S1; *T*: tubular atrophy/interstitial

fibrosis < 25% (T0), 26–50% (T1), or > 50% (T2); *C*: cellular/fibrocellular crescents absent (C0), present in at least 1

glomerulus (C1), in > 25% of glomeruli (C2)

^a^Time interval between renal biopsy of patients with IgA nephropathy and sample collection

^b^eGFR was calculated according to the Chronic Kidney Disease Epidemiology Collaboration (CKD-EPI) equation [19]

^c^CKD stages 1–5 were divided by eGFR≥90 (G1), 60–89 (G2), 45–59 (G3a), 30–44 (G3b), 15–29 (G4), and < 15 (G5) mL/min/1.73 m^2^, respectively, according to the new KDIGO (Kidney Disease: Improving Global Outcomes) classification [20]

^d^Determined in accordance with the Oxford classification [5]

### Serum levels of TNF-α in patients with IgAN and controls

**Fig. 1:**
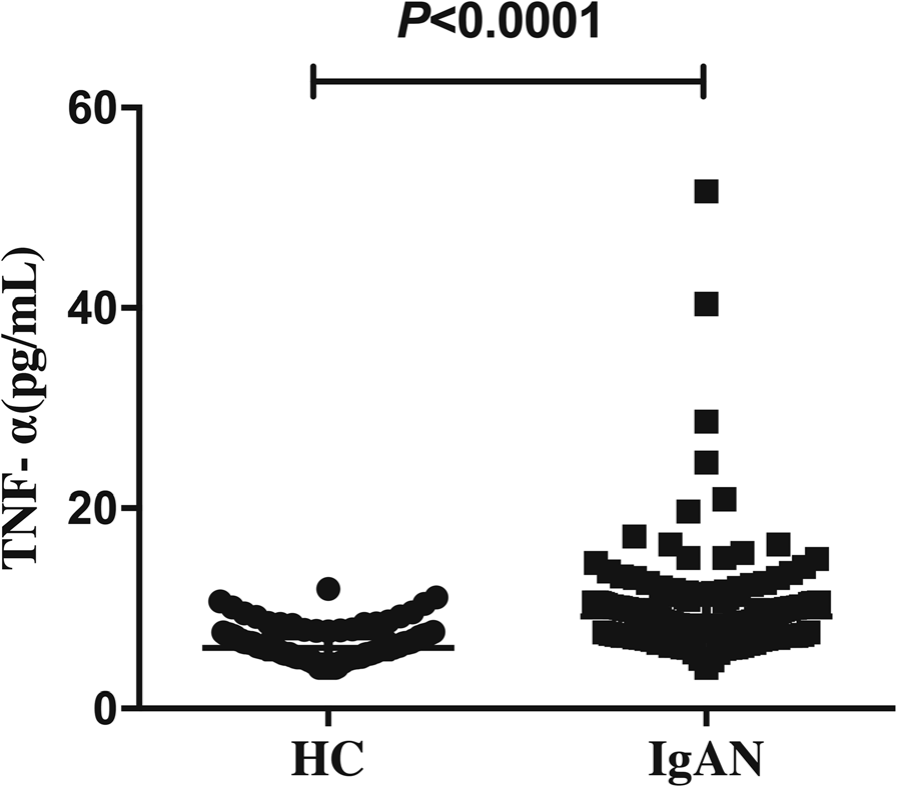
Serum levels of tumor necrosis factor alpha (TNF-α) between healthy controls (HC) and IgA nephropathy (IgAN) patients. There was a significant difference in serum levels of TNF-α [9.20 (7.70–10.60) pg/mL vs. 6.04 (5.11–7.23) pg/mL, *P* < 0.0001] between IgAN group (*n* = 147) and HC group (*n* = 126). Data are presented as median (interquartile range). Statistically significant differences between IgAN and HC were tested with Mann–Whitney U-test

As showed in Fig. 1, serum levels of TNF-α [9.20 (7.70–10.60) pg/mL vs. 6.04 (5.11–7.23) pg/mL, *P* < 0.0001] were significantly higher in IgAN group than that in HC group.

### Prediction values of TNF-α in patients with IgAN

**Fig. 2:**
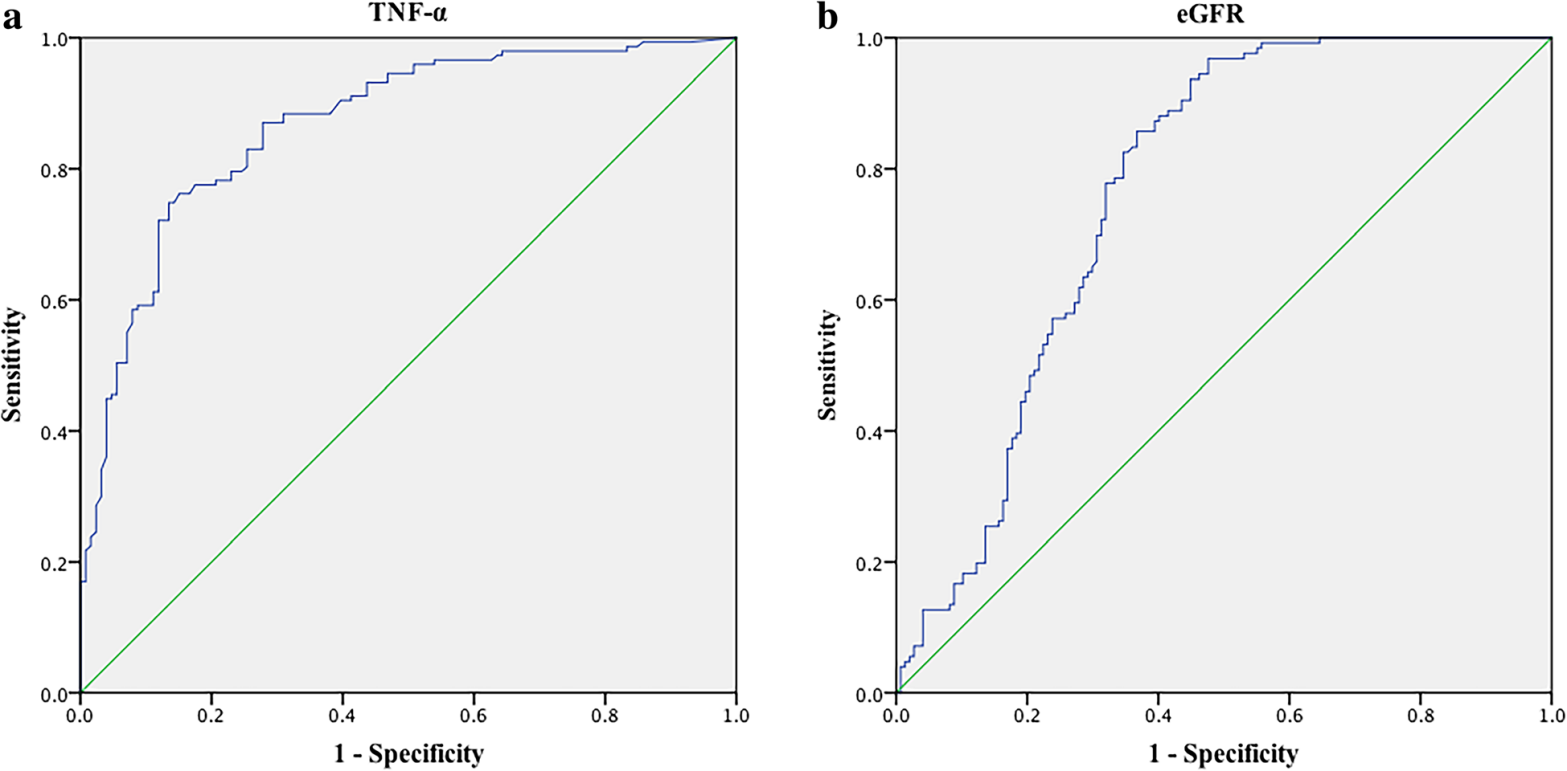
Receiver operating characteristic (ROC) curve analysis of the prediction values of tumor necrosis factor alpha (TNF-α) in IgA nephropathy (IgAN) patients. ROC curve revealed that **(a)** serum TNF-α had better discrimination than **(b)** eGFR between IgAN patients and healthy subjects [TNF-α: (AUC, 0.87; 95% CI, 0.83–0.91; *P* < 0.0001) vs. eGFR: (AUC, 0.76; 95% CI, 0.71–0.82; *P* < 0.0001), *P* = 0.007]. AUC, area under the curve; 95% CI, 95% confidence interval; Estimated glomerular filtration rate (eGFR) was calculated according to the Chronic Kidney Disease Epidemiology Collaboration (CKD-EPI) equation [19]

ROC analysis confirmed that TNF-α had better discrimination between patients with IgAN and healthy controls (HC) than eGFR [TNF-α: (AUC, 0.87; 95% CI, 0.83–0.91; *P* < 0.0001) vs. eGFR: (AUC, 0.76; 95% CI, 0.71–0.82; *P* < 0.0001), *P* = 0.007] (Fig. 2a and Fig. 2b). The respective optimal derived cut-off values were 7.79 pg/mL (Sensitivity: 74.8%; Specificity: 86.5%) for TNF-α, 80.36 mL/min•1.73m^2^ (Sensitivity: 96.8%; Specificity: 52.4%) for eGFR.

### Comparison of clinical and pathological features between patients with and without elevated serum TNF-α

We divided the patients into two subgroups based on the reference range of TNF-α which was established by the clinical laboratory in our hospital. The recommended reference range of serum TNF-α was from nondetectable to 8.1 pg/mL. Among 147 patients with IgAN, 98 patients were with elevated serum TNF-α and 49 patients were without elevated serum TNF-α. As showed in Table 2, compared with patients without elevated serum levels of TNF-α, patients with elevated serum levels of TNF-α had older age (*P* = 0.03) and higher proportion of male patients (*P* = 0.047). Significantly higher levels of systolic BP (*P* = 0.001), diastolic BP (*P* = 0.02), mean arterial pressure (*P* = 0.003), 24-h urine protein excretion (*P* = 0.03), urinary protein to serum creatinine ratio (*P* = 0.009), serum creatinine (*P* < 0.0001), Cystatin C (*P* < 0.0001) and lower levels of eGFR (*P* < 0.0001) were showed in patients with elevated serum levels of TNF-α. Proportions of mesangial hypercellularity (*P* = 0.04), segmental glomerulosclerosis (*P* = 0.02) and tubular atrophy/interstitial fibrosis grade (*P* = 0.008) were significantly higher in patients with elevated TNF-α than that in patients without elevated TNF-α.

**Table 2:** Demographic, clinical and pathological features of patients with and without elevated serum TNF-α

Characteristics	Subgroups		*P* Value
	TNF-α ≤ 8.1 pg/mL	TNF-α > 8.1 pg/mL	
Patients numbers	49	98	
Cytokines–biopsy time interval^a^ (months)	25.93 (5.27–55.90)	28.55 (7.14–67.47)	0.63
Mean age (yr)	33 (28–41)	40 (29–47)	0.03
Male, n (%)	18 (36.7)	53 (54.1)	0.047
Systolic BP (mmHg)	120 (110–120)	120 (118–130)	0.001
Diastolic BP (mmHg)	70 (60–80)	80 (70–80)	0.02
Mean arterial pressure (mmHg)	87 (78–93)	93 (87–97)	0.003
Urinary red blood cell (/μL)	34 (7–131)	22 (7–67)	0.13
Urinary protein to serum creatinine ratio (mg/g Cr)	486 (159–881)	756 (335–1596)	0.009
24-h urine protein excretion (g/24 h)	0.68 (0.30–1.09)	0.99 (0.40–1.70)	0.03
< 0.3	12 (24.5%)	15 (15.3%)	0.009
0.3–0.99	23 (46.9%)	36 (36.7%)	
1.0–2.99	13 (26.5%)	33 (33.7%)	
≥ 3	1 (2.0%)	14 (14.3%)	
Serum creatinine (mg/dL)	0.89 (0.72–1.07)	1.19 (0.92–1.52)	< 0.0001
Cystatin C (mg/dL)	0.98 (0.79–1.12)	1.22 (1.00–2.00)	< 0.0001
eGFR (mL/min•1.73m^2^)^b^	92.95 (73.96–118.78)	70.94 (52.26–91.57)	< 0.0001
CKD Stages^c^, n (%)			< 0.0001
1	30 (61.2)	27 (27.6)	
2	15 (30.6)	34 (34.7)	
3a	2 (4.1)	20 (20.4)	
3b	1 (2.0)	7 (7.1)	
4	1 (2.0)	4 (4.1)	
5	0 (0.0)	6 (6.1)	
Oxford classification^d^, n (%)			
M0/ M1	9 (18.4), 40 (81.6)	7 (7.1),91 (92.9)	0.04
E0/E1	38 (77.6), 11 (22.4)	75 (76.5), 23 (23.5)	0.89
S0/S1	13 (26.5), 36 (73.5)	11 (11.2), 87 (88.8)	0.02
T0/T1/T2	22 (44.9), 19 (38.8), 8 (16.3)	23 (23.5), 46 (46.9), 29 (29.6)	0.008
C0/C1/C2	22 (44.9), 23 (46.9), 4 (8.2)	44 (44.9), 43 (43.9), 11 (11.2)	0.79
Medical treatments, n (%)			
Untreated	10 (20.4)	25 (25.5)	0.49
Corticosteroids	22 (44.9)	49 (50.0)	0.56
Immunosuppressants	26 (53.1)	50 (51.0)	0.82
ACE-Is/ARBs	34 (69.4)	65 (66.3)	0.71

Data are presented as median (interquartile range) or frequency in percent

*BP*: blood pressure; 1 mmHg = 0.133Kpa; *eGFR*: estimated glomerular filtration rate; *M*: mesangial hypercellularity score < 0.5 (M0) or > 0.5 (M1); E: endocapillary hypercellularity absent (E0) or present (E1); *S*: segmental glomerulosclerosis absent (S0) or present (S1), presence or absence of podocyte hypertrophy/tip lesions in biopsy specimens with S1; *T*: tubular atrophy/interstitial fibrosis < 25% (T0), 26–50% (T1), or > 50% (T2); *C*: cellular/fibrocellular crescents absent (C0), present in at least 1 glomerulus (C1), in > 25% of glomeruli (C2)

^a^Time interval between renal biopsy of patients with IgA nephropathy and sample collection

^b^eGFR was calculated according to the Chronic Kidney Disease Epidemiology Collaboration (CKD-EPI) equation [19]

^c^CKD stages 1–5 were divided by eGFR≥90 (G1), 60–89 (G2), 45–59 (G3a), 30–44 (G3b), 15–29 (G4), and < 15 (G5) mL/min/1.73 m^2^, respectively, according to the new KDIGO (Kidney Disease: Improving Global Outcomes) classification [20]

^d^Determined in accordance with the Oxford classification [5]

### Correlations between serum levels of TNF-α and clinical features of patients with IgAN

Significant higher serum levels of TNF-α were observed in male patients with IgAN than that in female patients [9.70 (8.10–11.10) pg/mL vs. 8.50 (7.20–10.38) pg/mL, *P* = 0.02]. Serum levels of TNF-α were significantly positively correlated with systolic BP (*r* = 0.30, *P* < 0.0001), diastolic BP (*r* = 0.19, *P* = 0.02), 24-h urine protein excretion (*r* = 0.22, *P* = 0.007), urinary protein to serum creatinine ratio (*r* = 0.26, *P* = 0.002), serum creatinine (*r* = 0.49, *P* < 0.0001) and Cystatin C (*r* = 0.51, *P* < 0.0001) in IgAN. And serum levels of TNF-α were negatively correlated with eGFR (*r* = − 0.47, *P* < 0.0001) in bivariate correlation analysis (Table 3). Serum levels of TNF-α was not significantly correlated with age, serum IgA levels or urinary red blood cells count. Multivariate linear regression analyses showed that serum levels of TNF-α were positively correlated with 24-h urine protein excretion (*r* = 0.33, *P* = 0.04), urinary protein to serum creatinine ratio (r = 0.33, *P* = 0.03), serum creatinine (*r* = 0.46, *P* < 0.0001) and Cystatin C (*r* = 0.59, *P* < 0.0001) in IgAN and negatively correlated with eGFR (*r* = − 0.49, *P* < 0.0001) after adjustment for sex, systolic BP and diastolic BP (Table 3).

**Table 3:** Correlation between serum TNF-α and clinical parameters of patients with IgA nephropathy

Variable	Bivariate Regression Coefficient	Bivariate *P* Value ^a^	Multivariate Regression Coefficient	Multivariate *P* Value ^b^
Sex		0.02 ^c^		
Systolic BP	0.30	< 0.0001		
Diastolic BP	0.19	0.02		
24-h urine protein excretion	0.22	0.007	0.33	0.04
Urinary protein to serum creatinine ratio	0.26	0.002	0.33	0.03
Serum creatinine	0.49	< 0.0001	0.46	< 0.0001
Cystatin C	0.51	< 0.0001	0.59	< 0.0001
eGFR ^d^	−0.47	< 0.0001	0.49	< 0.0001

*BP*: blood pressure; 1 mmHg = 0.133Kpa; *eGFR*: estimated glomerular filtration rate

^a^Spearman's correlation analysis unless otherwise specified

^b^Multiple linear regression adjusted for sex, Systolic BP and Diastolic BP

^c^Mann–Whitney U-test

^d^eGFR was calculated according to the Chronic Kidney Disease Epidemiology Collaboration (CKD-EPI) equation [19]

### Correlations between serum levels of TNF-α, and pathological features of patients with IgAN

**Fig. 3:**
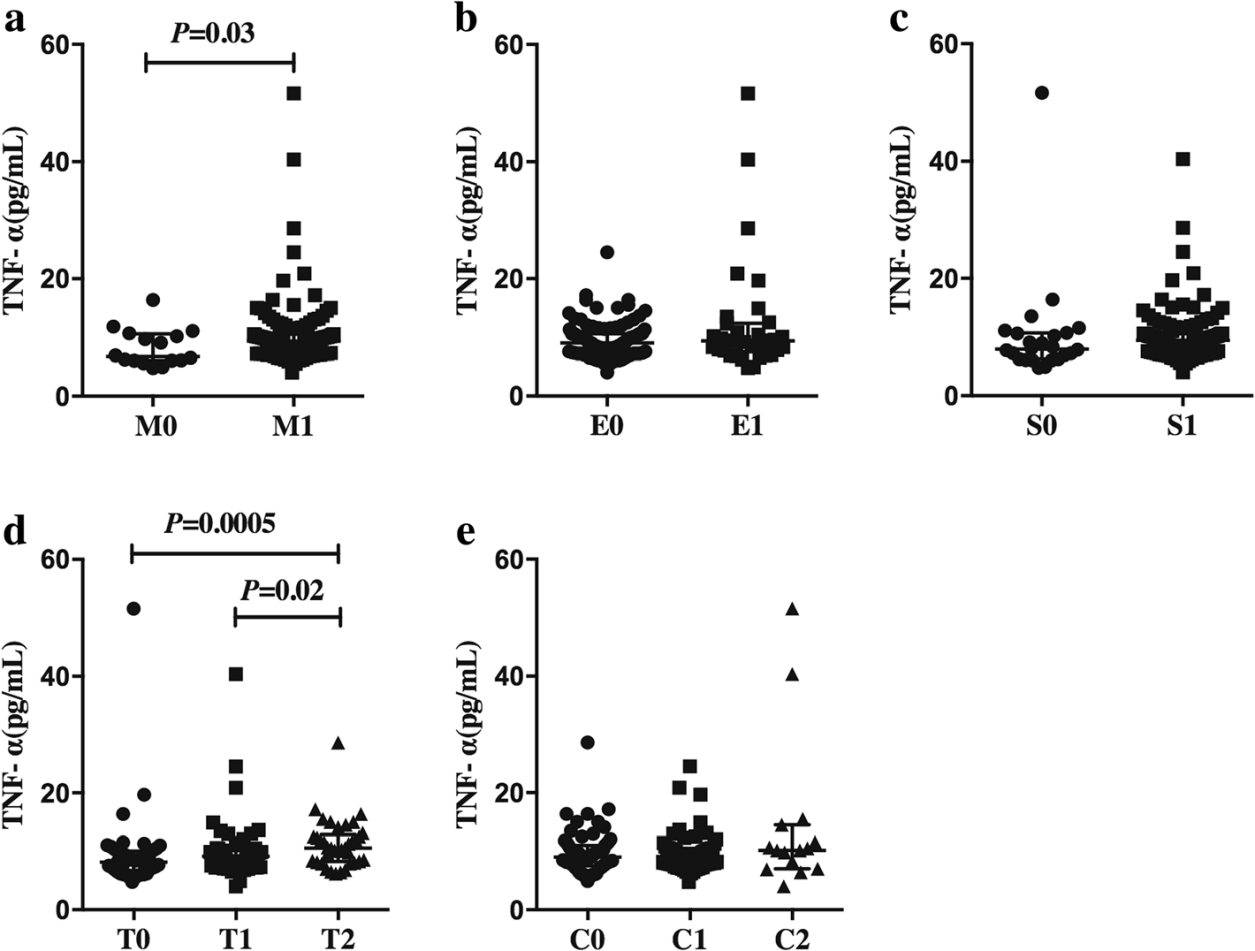
Correlations between serum levels of tumor necrosis factor alpha (TNF-α) and different grade according to Oxford Classification ^a^. (**a**) Serum levels of TNF-α were significantly higher in patients with mesangial hypercellularity grade M1 than that in patients with grade M0 [M1: 9.30 (7.90–10.60) pg/mL vs. M0: 6.70 (6.00–10.58) pg/mL, *P* = 0.03]. (**d**) Serum levels of TNF-α in patients with tubular atrophy/interstitial fibrosis grade T2 were significantly higher than that in grade T0 [T2: 10.60 (8.25–12.85) pg/mL vs. T0: 6.70 (6.00–10.58) pg/mL, *P* = 0.0005] and T1 [T2: 10.60 (8.25–12.85) pg/mL vs. T1: 9.20 (8.00–10.20) pg/mL, *P* = 0.02]; But there was no difference between patients with grade T1 and T0 (*P* = 0.05). (**b, c** and **e**) Serum levels of TNF-α in patients with different grades of endocapillary hypercellularity [E1: 9.35 (7.53–12.35) pg/mL vs. E0: 9.00 (7.75–10.60) pg/mL, *P* = 0.66], segmental glomerulosclerosis [S1: 9.40 (7.90–10.60) pg/mL vs. S0: 7.90 (6.20–10.68) pg/mL, *P* = 0.06] and cellular/fibrocellular crescents [C2:10.20 (7.00–14.50) pg/mL vs. C0: 9.00 (7.53–11.03) pg/mL, *P* = 0.22; C2: 10.20 (7.00–14.50) pg/mL vs. C1: 9.15 (7.80–10.50) pg/mL, *P* = 0.26; C1: 9.15 (7.80–10.50) pg/mL vs. C0: 9.00 (7.53–11.03) pg/mL, *P* = 0.81] did not show any significant difference. Statistically significant differences were tested with Mann–Whitney U-test. ^a^ Determined in accordance with the Oxford classification [5]

As showed in Fig. 3, IgAN patients with higher scores in the variable mesangial hypercellularity showed significant higher serum levels of TNF-α [M1: 9.30 (7.90–10.60) pg/mL vs. M0: 6.70 (6.00–10.58) pg/mL, *P* = 0.03]. Patients with higher scores in the variable tubular atrophy/interstitial fibrosis grade showed higher serum levels of TNF-α [T2:10.60 (8.25–12.85) pg/mL vs. T1: 9.20 (8.00–10.20) pg/mL vs. T0: 8.20 (6.70–10.05) pg/mL, overall *P* = 0.001]. Serum levels of TNF-α in patients with tubular atrophy/interstitial fibrosis grade T2 were significantly higher than that in grade T0 [T2:10.60 (8.25–12.85) pg/mL vs. T0: 6.70 (6.00–10.58) pg/mL, *P* = 0.0005] and T1 [T2: 10.60 (8.25–12.85) pg/mL vs. T1: 9.20 (8.00–10.20) pg/mL, *P* = 0.02]; But there was no difference between patients with grade T1 and T0 (*P* = 0.05). However, serum levels of TNF-α in patients with different scores of endocapillary hypercellularity, segmental glomerulosclerosis and cellular/fibrocellular crescents show no significant difference (*P* > 0.05).

## Discussion

TNF-α is a kind of proinflammatory cytokines that has been involved in certain forms of immune-mediated renal injury, including IgAN [21]. In the present research, we enrolled 147 IgAN patients and investigated the correlations between the serum levels of TNF-α detected by CLIA and the clinicopathological features. Results showed serum levels of TNF-α were significantly higher in patients with IgAN than in healthy subjects. The ROC analysis revealed that the optimal cut-off value of serum TNF-α had better discrimination between IgAN patients and healthy subjects than eGFR. In addition, serum TNF-α significantly correlated with urine protein, and renal function on both bivariate and multivariate linear regression analysis. And serum TNF-α also correlated with mesangial hypercellularity and tubular atrophy/interstitial fibrosis according to Oxford classification in IgAN.

D’Amico G has come the concordance that severe proteinuria, arterial hypertension and impairment of renal function were the strongest and most reliable clinical predictors for an unfavorable prognosis after critical analysis of results of 23 studies [4]. Mesangial hypercellularity and tubular atrophy/interstitial fibrosis lesion judged by updated Oxford classification are also independent predictors of long-term renal outcome [5]. Since serum levels of TNF-α were closely correlated with these three clinical risk factors and pathological features in the study, the results indicated serum levels of TNF-α was a biomarker for disease severity in IgAN.

Human recombinant TNF-α administered intravenously to rabbits induces endothelial damage, and leucocyte and fibrin accumulation in the glomerular capillary lumen [22]. In vitro, TNF-α stimulates the growth of epithelial glomerular cells in culture [23]. However, serum levels of TNF-α were not associated with endocapillary hypercellularity, segmental glomerulosclerosis and cellular/fibrocellular crescents in this study. Since nearly a half of our patients were treated with immunosuppressive therapy in this research, which possibly suppressed the production of cytokines, might interfere with the results. The damage of TNF-α in glomerular endothelial and epithelial cells except that in mesangial cells in IgAN should be determined [11, 12, 24].

Matsumoto K et al. had showed a large proportion of serum TNF originates from serum mononuclear cells, which synthesize increased amounts of TNF-α in IgAN [25]. Since exposure of microbial pathogens triggers the production of TNF-α by Toll-like receptors / myeloid differentiation primary response gene 88 pathways in IgAN [12, 26], TNF-α overproduction in mucosa may be a resource of serum TNF-α. As significantly higher serum levels of TNF-α indicated more severe clinicopathological manifestations and three IgAN patients not involved in this research with the levels of TNF-α higher than 80 pg/mL suffered macroscopic hematuria only hours after respiratory tract infection, we supposed that a direct mucosa-kidney talk that was mediated at least in part by TNF-α may exist [27, 28]. Later researches focusing the mucosa-kidney talk are needed.

To our acknowledgement, this is the first study measuring the serum levels of TNF-α by CLIA in patients with IgAN. Tumor necrosis factor – alpha Assay Kit (chemiluminescence) using in the present study to detect serum TNF-α is a novel and reliable method. The method comparison result shows that the linear relationship between CLIA and ELISA assay is 0.97 for measuring TNF-α. The most importantly, CLIA as a quantitative method which has higher sensitivity than semi-quantitative ELISA, and better stability and wider dynamic range for detecting TNF-α [18, 29]. Furthermore, CLIA is a fully automated technique which can save time, reduce intra- and inter-laboratory variability and improve the reproducibility of the results [18, 29]. Therefore, monitoring serum levels of TNF-α in patients with IgAN will be easy in clinical practice.

There were some limitations in our research. First, a well-designed study with appropriate follow-up period is needed to assess the correlation between serum levels of TNF-α and the renal outcomes in patient with IgAN. Second, variable time differences between renal biopsy and sample collection of the patients might affect the correlations between TNF-α and clinicopathological features. Different treatment regimens in the research might interfere with the results of cytokines. Although it may be tempting to speculate that the serum levels of TNF-α is a prognostic biomarker in IgAN patients, the cross-sectional nature of the study did not allow us to draw a definitive conclusion. This hypothesis may be tested in prospective investigations. Our 10-year cohort study determining whether serum levels of TNF-α is an independent prognostic factor recently approved by Ethics Committee and our randomized controlled study focusing on the therapeutic response of cytokines including TNF-α treated with HCQ in IgAN (ClinicalTrials.gov number, NCT02765594) are both in progress.

## Conclusions

We reported that the serum levels of TNF-α, detected by CLIA, were closely associated with disease severity in IgAN. Serum TNF-α was a potential biomarker for severity of patients with IgAN.

## Additional file

Additional file 1: Demographic and clinical parameters of healthy subjects. (DOCX 17 kb)

## Abbreviations

95% CI: 95% Confidence interval
ACE-Is: Angiotensin-converting enzymes inhibitors
ARBs: Angiotensin II receptor blockers
AUC: Area under the curve
BP: Blood pressure
C: Cellular/fibrocellular crescents
CKD: Chronic kidney disease
CKD-EPI: Chronic Kidney Disease Epidemiology Collaboration
CLIA: Chemiluminescence immunoassay
E: Endocapillary hypercellularity
EGF: Epidermal growth factor
eGFR: Estimated glomerular filtration rate
ELISA: Enzyme linked immunosorbent assay
HC: Healthy controls
HCQ: Hydroxychloroquine
IgAN: IgA nephropathy
IL-6: Interleukin-6
LN: Lupus nephritis
M: Mesangial hypercellularity
MCD: Minimal change disease
MN: Membranous nephropathy
ROC: Receiver operating characteristic
S: Segmental glomerulosclerosis
T: Tubular atrophy/interstitial fibrosis
TNF-α: Tumor necrosis factor alpha
