# Supplementary material for: Serum levels of tumor necrosis factor alpha in patients with IgA nephropathy are closely associated with disease severity
Source: BMC Nephrol. 2018 Nov 14;19:326. doi: 10.1186/s12882-018-1069-0 (PMC6236996; doi:10.1186/s12882-018-1069-0)
Supplement: Supplementary file 1 — Demographic and clinical parameters of healthy subjects. (DOCX 17 kb) [file 12882_2018_1069_MOESM1_ESM.docx]

**Table S1.** Demographic and clinical parameters

of healthy subjects

| Characteristics | |  | Values |
| --- | --- | --- | --- |
| Patients numbers |  |  | 126 |
| Mean age (yr) |  |  | 40 (30-49) |
| Male, n (%) |  |  | 61 (48.4%) |
| Systolic BP (mmHg) | |  | 119(109-130) |
| Diastolic BP (mmHg) | |  | 75(67-81) |
| Mean arterial pressure (mmHg) | |  | 89(81-97) |
| Serum creatinine (mg/dL) | |  | 0.81(0.70-0.92) |
| eGFR (mL/min•1.73m^2^) ^a^ | |  | 108.24 (95.45-118.97) |
| ~~Serum albumin (g/L)~~ |  |  | ~~46(45-48)~~ |

Data are presented as median (interquartile range) or frequency in percent.

BP: blood pressure; 1mmHg=0.133Kpa; eGFR: estimated glomerular filtration rate

^a^ eGFR was calculated according to the Chronic Kidney Disease Epidemiology Collaboration (CKD-EPI) equation [1]

**References**

1. Stevens LA, Claybon MA, Schmid CH, Chen J, Horio M, Imai E *et al*: Evaluation of the Chronic Kidney Disease Epidemiology Collaboration equation for estimating the glomerular filtration rate in multiple ethnicities. Kidney international 2011, 79(5):555-62.
